# How early microbial encounters shape adult behavior

**DOI:** 10.1371/journal.pbio.3004101

**Published:** 2026-10-30

**Authors:** Kyu-Chan Cho, Won-Jae Lee

**Affiliations:** School of Biological Sciences, Seoul National University, Seoul, Republic of Korea

## Abstract

Microbes are increasingly recognized as modulators of host behavior, but the underlying mechanisms are poorly understood. This Primer discusses a new PLOS Biology study that reveals a microbiome-to-gut-to-neuron pathway linking developmental microbial exposure to later food avoidance.

## Teaser

Microbes are increasingly recognized as modulators of host behavior, but the underlying mechanisms are poorly understood. A new *PLOS Biology* study reveals a microbiome-to-gut-to-neuron pathway linking developmental microbial exposure to later food avoidance.

Animals use sensory information to distinguish favorable environments from potential threats, such as harmful microbes, but these preferences are not determined by the genome alone. Early-life exposure to specific environmental cues can shape later behaviors, including feeding decisions, a phenomenon often referred to as priming [1,2]. However, the molecular mechanisms underlying such developmental priming remain poorly understood. According to a new study in *PLOS Biology* [3], Milleville and colleagues use *Drosophila* to show how oral exposure to a bacterial metabolite during the juvenile stage is translated into an adult feeding decision (Fig 1).

**Fig 1:**
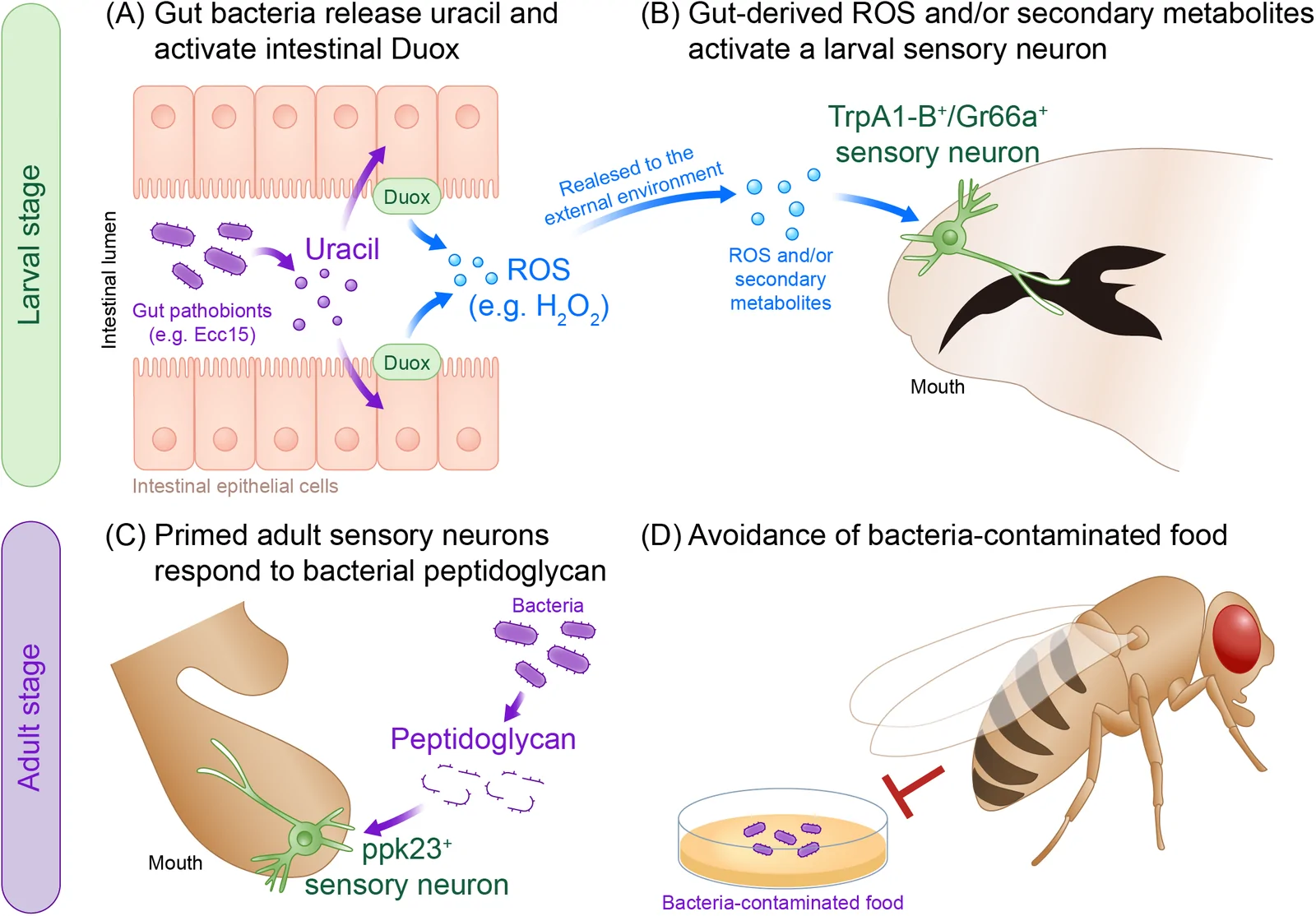
Early-life bacterial signals prime adult *Drosophila* feeding behavior through a gut–sensory neuron pathway. **(A)** In larvae, gut pathobionts release uracil into the intestinal lumen. Uracil activates Dual oxidase (Duox) in larval intestinal epithelial cells, including the production and luminal release of reactive oxygen species (ROS), including hydrogen peroxide. **(B)** Gut-derived ROS and/or secondary metabolites activate a TrpA1-B^+^/Gr66a^+^ sensory neuron in the larval terminal organ, an external sensory organ located at the mouth. **(C)** Following metamorphosis, adult flies exhibit a primed response to bacterial peptidoglycan that depends on ppk23^+^ sensory neurons at the mouth. How the larval signal persists through metamorphosis and influences this adult sensory response remains unknown. **(D)** Activation of ppk23^+^ sensory neurons promotes avoidance of bacteria-contaminated food.

The study builds on previous work from the same group showing that larvae fed certain gut microbes, particularly *Levilactobacillus brevis*, are primed as adults to avoid food containing peptidoglycan [2], a common component of bacterial cell walls. This effect requires larval gustatory neurons expressing Gustatory receptor 66a (Gr66a^+^ neurons) [2], best known for detecting bitter and aversive compounds [4]. These findings raised two key questions: what microbial signal triggers priming, and how is it detected by larval Gr66a^+^ neurons?

Milleville and colleagues first addressed the microbial signal. Larvae fed live *L. brevis* were primed to avoid peptidoglycan-containing food as adults, whereas those fed heat-killed bacteria or bacterial supernatant were not, suggesting that priming requires an actively produced microbial signal [3]. Previous work had shown that *L. brevis*, but not another common *Drosophila* gut bacterium, *Lactiplantibacillus plantarum*, induces priming [2]. Notably, *L. brevis*, unlike *L. plantarum,* can undergo a commensal-to-pathogen transition and thereby behave as a pathobiont—a normally harmless member of the microbiota that can transition to a pathogenic state under certain conditions [5]. This transition has been linked to bacterial uracil release through nucleoside catabolism [6], suggesting uracil as a candidate priming signal.

To test this possibility, the authors turned to *Erwinia carotovora* strain Ecc15 (also known as *Pectobacterium carotovorum*), a well-characterized *Drosophila* pathobiont for which strains differing in uracil release were available [6]. Larvae fed Ecc15 strains capable of releasing uracil developed adult peptidoglycan avoidance, whereas those fed strains defective in uracil release did not. Moreover, feeding larvae uracil alone was sufficient to prime adult peptidoglycan avoidance, whereas the related pyrimidine bases cytosine and thymine were ineffective [3]. Thus, uracil emerges as the bacterial cue that initiates developmental priming.

The authors next asked whether larval Gr66a^+^ neurons sense uracil directly or indirectly through a host-derived signal. Bacterial uracil is known to activate intestinal Dual oxidase (Duox), leading to production of reactive oxygen species (ROS) [5]. Uracil induced a transient increase in intestinal ROS, with hydrogen peroxide implicated as an important component, while genetic or pharmacological suppression of this redox response prevented priming [3]. Thus, Gr66a^+^ neurons appear to respond not directly to uracil, but to a host-generated redox signal induced by it.

How, then, is this host signal detected? Transient receptor potential ankyrin 1 (TrpA1), a sensory ion channel in larval Gr66a^+^ neurons, has previously been shown to be required for priming [2]. Among the different TrpA1 isoforms, the authors identified TrpA1-B as the isoform required in Gr66a^+^ neurons for this response. Silencing this neuronal population prevented adult peptidoglycan avoidance, whereas restoring TrpA1-B in Gr66a^+^ cells rescued priming in TrpA1-null animals [3]. Calcium imaging further showed that hydrogen peroxide activates this neuron in a TrpA1-dependent manner [3]. Together, these findings show that a bacterial signal triggers a host-derived signal, which in turn activates a specific sensory neuron to initiate developmental priming.

Perhaps the most intriguing part of the story remains unresolved. Activation of Gr66a^+^ neurons during the larval stage is required to establish priming, yet these neurons are not required for peptidoglycan avoidance in adults [2]. Instead, adult peptidoglycan avoidance depends on a distinct population of ppk23^+^ sensory neurons [2], which are best known as contact chemosensory neurons involved in pheromone detection and courtship behavior [7]. How, then, is information detected by larval Gr66a^+^ neurons transmitted across development to influence the adult ppk23^+^ neuron-dependent response? This is particularly intriguing because metamorphosis extensively remodels the *Drosophila* nervous system [8], yet the consequence of larval sensory experience persists into adulthood. One possibility is that larval Gr66a^+^ neuronal activation induces persistent changes in neural circuits that survive metamorphosis. Alternatively, early microbial exposure may alter the developmental programming of adult sensory circuits, thereby modifying their responsiveness to bacterial signals. These possibilities could be distinguished by tracing the fate of larval Gr66a^+^ neurons and/or their downstream circuits through metamorphosis [9], and by investigating whether early microbial exposure activates signaling pathways (e.g., endocrine signaling) that influence the development and function of adult ppk23^+^ neurons. Such studies would help elucidate the mechanisms by which larval microbial experience shapes adult behavior. Given the broader sensory and behavioral roles of ppk23^+^ neurons, it will also be interesting to ask whether Gr66a^+^ neuron-dependent developmental priming selectively affects ppk23^+^ neuron-mediated peptidoglycan avoidance or more broadly alters other ppk23^+^ neuron-dependent behaviors, such as pheromone sensing and courtship [7].

Another important question is why such developmental priming might have been favored through evolution. Early microbial encounters may provide information about environmental conditions likely to be encountered again, allowing later behavior to reduce future risk. Whether priming actually protects adult flies from infection or improves fitness, however, remains unknown. Comparing pathogen burden, tissue damage, survival, and reproductive success between primed and unprimed adults could test this possibility.

Early sensory experiences can have lasting effects on adult behavior [1,2]. The study by Milleville and colleagues extends this principle to host–microbe interactions by showing how a microbial signal encountered during development is translated through host signaling and a defined sensory neuron into a persistent behavioral change [3]. With its powerful genetic tools and well-defined microbial associations, *Drosophila* provides a tractable system for uncovering how transient microbial encounters leave lasting effects on the nervous system and behavior.
